# The reduction of faecal calprotectin during exclusive enteral nutrition is lost rapidly after food re‐introduction

**DOI:** 10.1111/apt.15425

**Published:** 2019-07-25

**Authors:** Michael Logan, Clare M. Clark, Umer Zeeshan Ijaz, Lisa Gervais, Hazel Duncan, Vikki Garrick, Lee Curtis, Elaine Buchanan, Tracey Cardigan, Lawrence Armstrong, Caroline Delahunty, Diana M. Flynn, Andrew R. Barclay, Rachel Tayler, Elizabeth McDonald, Simon Milling, Richard K. Hansen, Konstantinos Gerasimidis, Richard K. Russell

**Affiliations:** ^1^ Civil Engineering, School of Engineering University of Glasgow Glasgow UK; ^2^ Human Nutrition, School of Medicine, Dentistry and Nursing University of Glasgow, Glasgow Royal Infirmary Glasgow UK; ^3^ Department of Paediatric Gastroenterology Royal Hospital for Children Glasgow UK; ^4^ Department of Paediatrics Crosshouse Hospital Kilmarnock UK; ^5^ Department of Paediatrics Wishaw General Hospital Wishaw UK; ^6^ Institute for Infection, Immunity and Inflammation University of Glasgow Glasgow UK

## Abstract

**Background:**

Faecal calprotectin decreases during exclusive enteral nutrition in children with active Crohn's disease. It is unknown how faecal calprotectin changes during food re‐introduction and the influence of maintenance enteral nutrition.

**Aims:**

To study changes to faecal calprotectin during exclusive enteral nutrition and at food reintroduction, and explore associations with maintenance enteral nutrition.

**Methods:**

Children with Crohn's disease were followed during exclusive enteral nutrition and during food‐reintroduction. Faecal calprotectin was measured before, at 33 and 54 days of exclusive enteral nutrition, and at 17, 52 and 72 days after food‐reintroduction. Maintenance enteral nutrition use was recorded with estimated weight food diaries. Data are presented with medians and Q1:Q3.

**Results:**

Sixty‐six patients started exclusive enteral nutrition and 41 (62%) achieved clinical remission (weighted paediatric Crohn's disease activity index <12.5). Baseline faecal calprotectin (mg/kg) decreased after 4 and 8 weeks of exclusive enteral nutrition (Start: 1433 [Q1: 946, Q3: 1820] vs 33 days: 844 [314, 1438] vs 54 days: 453 [165, 1100]; *P* < .001). Within 17 days of food reintroduction, faecal calprotectin increased to 953 [Q1: 519, Q3: 1611] and by 52 days to 1094 [660, 1625] (both *P* < .02). Fifteen of 41 (37%) children in remission used maintenance enteral nutrition (333 kcal or 18% of energy intake). At 17 days of food reintroduction, faecal calprotectin was lower in maintenance enteral nutrition users than non‐users (651 [Q1: 271, Q3: 1781] vs 1238 [749, 2102], *P* = .049) and correlated inversely with maintenance enteral nutrition volume (rho: −0.573, *P* = .041), kcals (rho: −0.584, *P* = .036) and % energy intake (rho: −0.649, *P* = .016). Maintenance enteral nutrition use was not associated with longer periods of remission (*P* = .7). Faecal calprotectin at the end of exclusive enteral nutrition did not predict length of remission.

**Conclusions:**

The effect of exclusive enteral nutrition on faecal calprotectin is diminished early during food reintroduction. Maintenance enteral nutrition at ~18% of energy intake is associated with a lower faecal calprotectin at the early phase of food reintroduction but is ineffective in maintaining longer term remission.

## 1 INTRODUCTION

Crohn's disease (CD) is a chronic inflammatory condition which can affect any part of the gastrointestinal tract from mouth to anus. Symptoms include abdominal pain, bloody diarrhoea, weight loss, along with additional extra‐intestinal complications such as anaemia, skin rashes and arthritis.^1^

As Crohn's disease remains incurable, current therapy aims to induce and maintain clinical remission and, when possible, intestinal mucosal and transmural healing.^2^ Exclusive enteral nutrition (EEN) is a liquid‐only formula diet which is highly effective in achieving clinical remission in paediatric Crohn's disease.^3^ EEN is recommended as the first line treatment for active luminal Crohn's disease in children with up to 89% of patients achieving clinical remission when placed on EEN for 8 weeks.^4^, ^5^ Reviews and meta‐analyses have shown that EEN is as effective as oral corticosteroids in inducing clinical remission, in paediatric patients. It also has the added benefits of mucosal healing in a significant proportion of patients plus improvement in the overall nutritional status of patients.^6^, ^7^, ^8^, ^9^ Achieving complete mucosal healing after a course of EEN has been associated with reduced relapse rates and complications in the medium term.^2^

While the use of enteral nutrition as induction therapy of active Crohn's disease is well established, use of partial enteral nutrition, as a maintenance therapy (maintenance enteral nutrition or MEN), is less well studied.^10^ Previous research suggested that MEN may be effective in prolonging periods of clinical remission.^11^, ^12^, ^13^, ^14^, ^15^, ^16^, ^17^, ^18^, ^19^, ^20^, ^21^, ^22^ However, these studies have generally been of retrospective design, lacked robust assessment of MEN compliance and have not examined changes in gut markers of colonic inflammation.

We have previously described the effect that EEN has on faecal calprotectin, in children with active Crohn's disease during induction treatment with EEN, which has subsequently been replicated by others.^23^, ^24^ EEN reduces the pre‐treatment concentration of faecal calprotectin by an average of 48% nevertheless, less than 20%‐25% of those who have completed a successful course will have a measurement of FC below 250 mg/kg.^24^ In our previous research we also have described that faecal calprotectin levels increased rapidly in patients within 4 months of food reintroduction.^23^ Hence, it is of interest to study how quickly faecal calprotectin rises during the early and medium phase of food reintroduction in patients who completed a successful course of EEN, and whether MEN use can modify this effect. Furthermore, the concurrent change in cytokines paralleling changes in clinical and biochemical parameters has only been described in a few studies, contrasting with the well‐documented changes in patients undergoing biologic treatment.^25^, ^26^, ^27^, ^28^ The aim of the current study was to determine short and medium‐term changes in faecal calprotectin during food reintroduction, following induction of remission with EEN, and to explore the effectiveness of MEN to influence these changes in addition to risk of subsequent clinical relapse.

## 2 METHODS

### 2.1 Patients

Patients with suspected IBD who attended the Royal Hospital for Children, Glasgow and neighbouring district general hospitals, were recruited prospectively between October 2014 and May 2017. Diagnosis was based on established radiological, histological and endoscopic guidelines.^29^ Disease behaviour and anatomical location were classified using the Paris classification.^30^ Patients who were subsequently diagnosed with Crohn's disease were followed up and samples were collected throughout their EEN course and during food re‐introduction. Patients who had already received a diagnosis of Crohn's disease and were undergoing a repeat course of EEN were also recruited. Patients who had received antibiotics in the preceding month were excluded. Written assent or consent was taken from participants and their carers according to good clinical practice. The study was approved by the NHS West of Scotland Research Ethics Committee (14/WS/1004) and was registered in clinicaltrials.gov (NCT02341248).

### 2.2 Exclusive enteral nutrition and food re‐introduction

Patients were treated for 8 weeks with a polymeric, casein‐based formula (Modulen IBD). Paediatric dieticians calculated the feed volume to provide the energy requirements for each child based on the UK age and sex recommendations.^31^ Children who were undernourished (BMI Z‐score < −2 SD) were prescribed a 10% higher energy intake while on EEN. No other food was allowed during the EEN course, with the exception of black tea or coffee, water, or a sodium‐benzoate free carbonated sugar‐containing lemon and lime soft drink (7UP), clear mints and syrup flavouring. Patients were encouraged to consume EEN orally, however, if patients were unable to take their prescribed formula volumes within the first 3 days, patients were then switched to nasogastric tube feeding. While on EEN, patients received regular nurse and dietetic weekly support calls and were reviewed in the clinic 4 weeks post initiation. If the participant did not clinically improve or deteriorated within the initial treatment period, EEN therapy was discontinued and an alternative induction treatment was commenced, usually oral corticosteroids.

Patients who successfully completed their course of EEN and entered clinical remission (defined as a decrease in weighted paediatric Crohn' wPCDAI <12.5) were encouraged to continue using their enteral formula as an oral supplement (MEN), consuming approximately 20%‐25% of their total EEN volume, in addition to their free habitual diet. Patients had a rapid food reintroduction, with no specific dietary recommendations given by the members of the clinical team after the end of EEN, other than to offer MEN.

### 2.3 Clinical assessment

Measurements of anthropometry were obtained and calculated as Z‐scores using the UK‐WHO growth charts.^32^ C‐reactive protein (CRP), erythrocyte sedimentation rate (ESR), serum albumin and haemoglobin were measured at EEN initiation, end of treatment and at routine clinic appointments post‐EEN. Clinical disease activity was defined using the weighted paediatric Crohn's disease activity index (wPCDAI).^33^ Use of maintenance drug therapies were also recorded.

### 2.4 Faecal calprotectin measurements

Fresh faecal samples to measure faecal calprotectin were collected from children at enrolment, before they began treatment with EEN. Two subsequent samples were scheduled to be collected during patient's treatment with EEN at 30 and 56 days. In patients achieving clinical remission, two further samples were collected; the first around 2 weeks (15 days) post‐EEN and the second around their 2‐month (56 days) scheduled clinic review. If a sample was not collected at clinic review, patients were asked to provide a sample at their next time of convenience. Patients collected the whole bowel movement in a plastic container. The sample was collected within 3.7 hours (Q1: 2.9, Q3: 5.0) of defecation and homogenised by mechanical kneading before aliquots were stored at −70°C until further analysis was performed. Faecal calprotectin concentrations were determined using the CALPROLAB0170 (ALP) (Lysaker, Norway) ELISA kit according to the manufacturer's instructions and as described previously.^34^

### 2.5 Cytokine profile changes during EEN

Blood samples were collected in EDTA tubes and plasma separated within 2 hours. The concentration of 19 inflammatory cytokines was analysed using the U‐PLEX Proinflam Combo 1 (IFN‐γ, IL‐10, IL‐12p70, IL‐13, IL‐1β, IL‐2, IL‐4, IL‐6, IL‐8, TNF‐α) (K15049K) and the U‐PLEX TH17 Combo (IL‐17A, IL‐17E/IL‐25, IL‐17F, IL‐21, IL‐22, IL‐23, IL‐27, IL‐31, IL‐33) (K15075K) assay kits (Meso Scale Discovery).

### 2.6 Dietary assessment

During food re‐introduction and prior to sample collection, at 2 weeks and 2 months post‐EEN, the energy intake of participants was estimated using 3‐day estimated weight food diaries. Household measures were used to estimate portion size. Patients were asked to record compliance with MEN and the exact amount consumed on the same food diaries. These diaries were analysed into WinDiets 10 Software Suite (version 10; Robert Gordon University), to calculate energy intake.

Associations were explored between faecal calprotectin and both the volume and energy intake attributable to MEN. Energy intake was expressed in kcals, as percentage of patient's estimated average energy requirement (EAR) and as a percentage of energy intake. No MEN intake was assigned to patients who returned a food diary without reporting use of MEN.

### 2.7 Rates of remission and relapse within 1‐year post EEN

Patients were followed up to a maximum of 12 months, following completion of EEN. Clinical relapse was defined as deterioration of disease requiring a further induction therapy as dictated by the clinical caring team.

### 2.8 Statistics

Continuous data are displayed with median and interquartile range, unless otherwise stated. Differences in rates of remission based on disease location were evaluated using a χ^2^ test. Two‐sample *t* tests were performed to compare differences between two independent groups in patient characteristics. Mann‐Whitney *U* tests were performed to explore differences in proportional changes in faecal calprotectin during food reintroduction grouped together using various cut‐offs of faecal calprotectin at the end of EEN. Serial changes to faecal calprotectin during EEN were tested using a general linear model accounting for longitudinal measurements from the same subject. Post‐hoc comparisons were performed using the Fisher least significant difference correction. A further general linear model, accounting for the days between sample collection, was performed to assess whether faecal calprotectin concentrations at the end of EEN could predict the subsequent rise in FC during food reintroduction.

Receiver operating curves (ROC) were used to estimate the ability of percentage change in faecal calprotectin, during the first 4 weeks of treatment to predict patients entering clinical remission by the end of EEN. Sensitivity, specificity, positive and negative predictive values were calculated.

Kaplan‐Meier curves with Mantel‐Cox log rank test was used to perform survival analysis on the time to relapse based on the use of MEN, as taken from patient's food diaries. The same analysis was also performed using various faecal calprotectin cut‐offs at the end of EEN to predict the time to subsequent clinical relapse.

Statistical analysis was performed using Minitab version 18 statistical software (Minitab Ltd). *P*‐values below .05 were considered statistically significant.

## 3 RESULTS

### 3.1 Patient population

Sixty‐eight children with Crohn's disease (25 [37%] females, median age 13.4 [Q1: 10.4, Q3: 14.6] years) were recruited. Six out of 68 (9%) patients had a previous Crohn's disease diagnosis and were undergoing a second course of EEN with the remainder being newly diagnosed and treatment naive. Twenty‐five (37%) children had panenteric disease; 13 (19%) presented with isolated colonic disease; and four (6%) had isolated ileitis. All patients presented with inflammatory (B1) disease behaviour (Table 1). At treatment initiation, patients presented with a median BMI z‐score of −0.73 (Q1: −1.68, Q3: 0.21); 13 (19%) patients were classed as thin (BMI z‐score < −2) and, no patients were obese. Median height z‐score was −0.27 (Q1: −0.7, Q3: −0.43) with 5 (8%) children classed as short stature (Height z‐score < −2SD).

**Table 1:** Baseline demographics and phenotypic characteristics of the 66 Crohn's disease patients treated with exclusive enteral nutrition

	N (%)
Males (%)	42 (64)
Newly diagnosed (%)	60 (91)
Age (y)	13.4 (10.7, 14.9)
Age at diagnosis (%)	
A1a (0 to <10)	14 (21.2)
A1b (10 to <17)	50 (75.8)
A2 (17 to <40)	2 (3)
Disease location (%)	
L1, B1	4 (6)
L1, L4a, B1	2 (3)
L2, B1	11 (17)
L2, L4a, B1	10 (15)
L2, L4a, L4b, B1	3 (5)
L3, B1	9 (14)
L3, L4a, B1	10 (15)
L3, L4a, L4b, B1	14 (21)
L3, L4b, B1	2 (3)
L4a, B1	1 (2)
Perianal	6 (9)
Disease behaviour (%)	
B1	66 (100)
B2	0
B2	0
B2B3	0

Note:

Paris disease classification.

In the 53 children who completed EEN, BMI z‐scores significantly improved (EEN start: −0.67 [−1.52, 0.29], 54 days EEN: −0.04 [−0.46, 0.57], *P* < .001) with a median weight gain of 2.4 kg (Q1: 0.83, Q3: 4.44) over the 8‐week course of EEN (Table S1).

**Figure 1:**
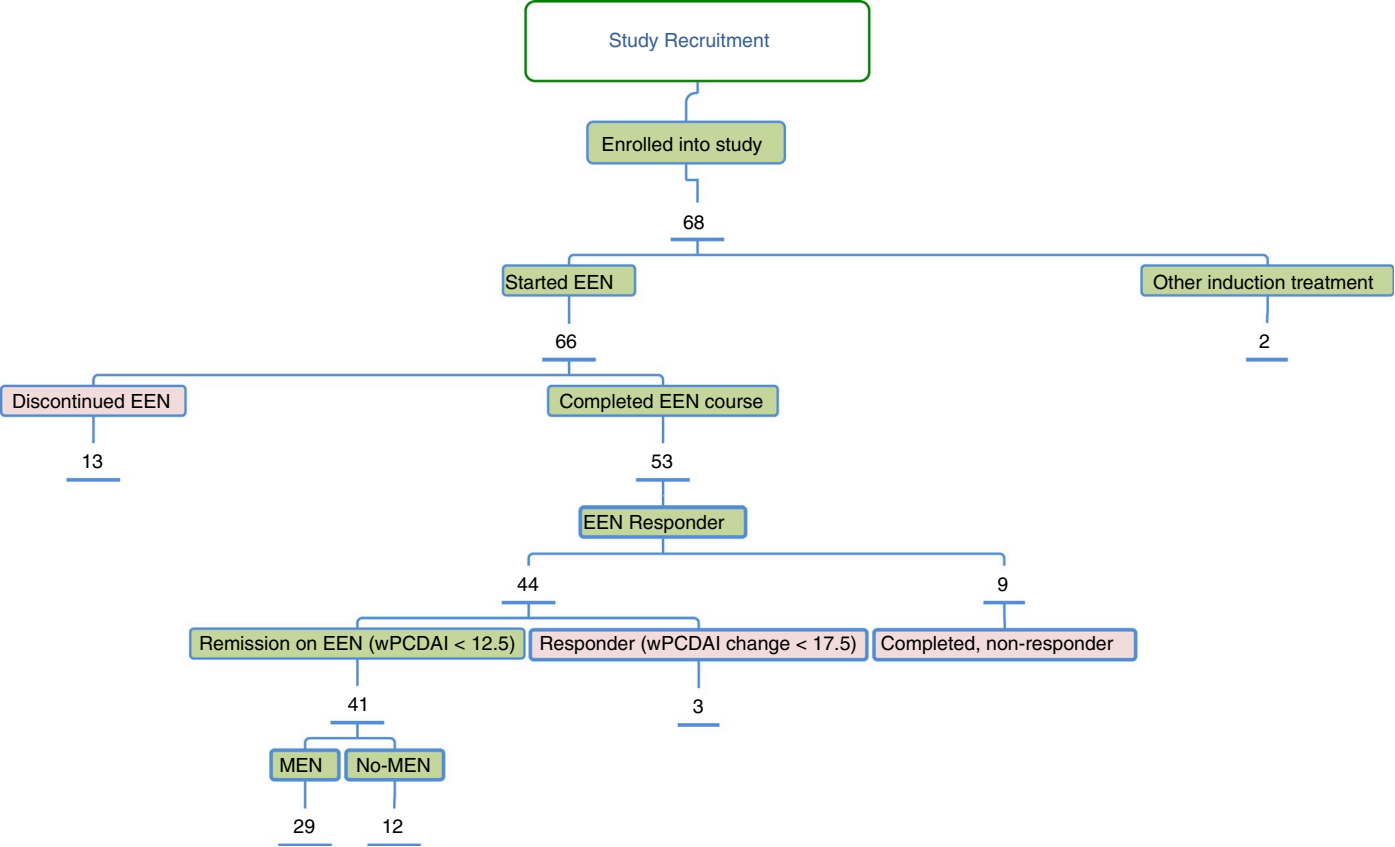
Schematic flowchart of patients recruited to study with treatment outcomes during EEN (response defined by weighted paediatric Crohn's disease activity index). Abbreviations: EEN, exclusive enteral nutrition

Sixty‐six (97%) patients were initially treated with EEN, with one newly diagnosed patient starting directly on anti‐TNF therapy, and another with mild symptoms starting treatment with budesonide enemas. Of the 66 patients to start EEN, 53 (80%) completed at least 42 days (median, 55 days; Q1: 45, Q3: 56). Fourteen (14/66, [21%]) patients required nasogastric tube feeding, while the remaining 52/66 (79%) consumed EEN orally. Clinical remission was achieved in 41 (62%) patients (Figure 1). Remission on EEN was independent of disease location (*P* = 0.25) (Table S2). Of the 25 patients not to achieve remission on EEN, 13 discontinued treatment (due to intolerance or symptom deterioration). The other 12 children completed an 8‐week course of EEN; of whom three achieved clinical response and the remaining nine patients did not show clinical response using wPCDAI definitions.

### 3.2 Systemic inflammatory markers changes during EEN

Sixty‐three (63/66 [95%]) patients had systemic inflammatory markers available at treatment initiation of whom 40 (65%) had at least one inflammatory marker (CRP, ESR, albumin) abnormal. These inflammatory markers improved when patients completed EEN, with 12/40 (30%) having at least one marker outside the normal range. Among those who entered clinical remission or clinically improved, and whose blood measurements were available, 22/32 (69%) patients had all normal inflammatory makers (Table S1).

### 3.3 Faecal calprotectin concentration changes during EEN

From the 66 patients who started on EEN, a faecal sample was obtained for baseline faecal calprotectin measurement in 53 (80%). Median faecal calprotectin at the start of treatment was 1433 mg/kg [Q1: 947, Q3: 1820] with 52/53 having a raised faecal calprotectin (>50 mg/kg), and 50 (94%) having a value >250 mg/kg.

During EEN, a sample was collected at a median of 33 days (Q1: 36, Q3: 39), by which time faecal calprotectin concentrations had decreased by a median of 597 mg/kg from baseline values (33 days EEN concentration: 836 mg/kg [Q1: 296, Q3: 1303], *P* < .001; % decrease from baseline: 33% [−17.9, 76.2]). As patients completed EEN, a sample was collected at a median of 54 days (53, 56) on treatment. The reduction in faecal calprotectin continued during the second half of treatment and reached a median of 453 mg/kg (165, 1100; *P* < 0.001) compared with EEN start by the end of EEN; decreasing by a median of 59% (−3.2, 88.7) from their baseline value.

There was no difference in baseline faecal calprotectin levels between patients who achieved remission on EEN and those who did not (remission group, median 1447 mg/kg [855, 1839] vs non‐remission median 1684 mg/kg [1233, 1857]; *P* = .67). In patients who completed 8 weeks of EEN, there was no difference in faecal calprotectin at the end of EEN between patients achieving remission and those that did not (remission group FC: N = 29, 430 mg/kg [Q1: 141, Q3: 1047], no remission group FC: N = 8, 911 mg/kg [Q1: 370, Q3: 1791], *P* = .121). When performed without the requirement of completing EEN and instead using the patients ‘exit’ sample i.e. including samples that may be collected at 4wk EEN if the patient discontinued at this point then there is a significant difference in FC between remission vs no remission (remission: n = 35 median: 451 [133, 1068] vs no‐remission: n = 12 median: 1331 [497, 1596], *P* value = 0.04).

Patients who entered clinical remission by the end of EEN had a greater reduction in faecal calprotectin by the midpoint of EEN than those who did not achieve remission (remission group FC change: 689 mg/kg [246, 1127]; non‐remission group FC change: 182 mg/kg [−144, 548]; *P* = 0.007) (Table S1).

Patients who completed EEN without achieving remission did not have a significant decrease in faecal calprotectin by the end of treatment (EEN start: 1322 [1148, 1699], 54 days EEN: 1233 [265, 1732], *P* = .35).

Using ROC curve analysis, a percentage reduction in faecal calprotectin during the first 4 weeks of EEN resulted in an AUC of 0.75 (CI: 0.59‐0.92) and it was found that a reduction of greater than 45% from baseline concentrations produced a sensitivity of 100%, a 57.7% specificity and a positive and negative predictive validity of 100% and 38.9% of detecting patients who achieved remission at the end of EEN, respectively (Table 2). Similar ROC curve analysis was performed using changes in anthropometry, specifically the change in weight (kg), % weight change (kg), weight z‐score, during the same time period. These models returned AUC of 0.79 (CI: 0.65‐0.9); 0.76 (CI: 0.61‐0.9); 0.75 (CI: 0.57‐0.93) respectively.

**Table 2:** Prediction of clinical remission by 8 wk EEN, using % FC decrease from baseline to 4 wk EEN

% 0 to 4 wk FC decrease	Sensitivity (%)	95% CI	Specificity (%)	95% CI	Likelihood ratio	PPV (%)	NPV (%)
>19.2	42.9	9.9‐81.6	69.2	48.2‐85.7	1.39	81.8	27.3
>24.2	57.1	18.4‐90.1	69.2	48.2‐85.7	1.86	85.7	33.3
>29.3	71.4	29.0‐96.3	69.2	48.2‐85.7	2.32	90	38.5
>33.2	71.4	29.0‐96.3	65.4	44.3‐82.8	2.06	89.5	35.7
>34.0	71.4	29.0‐96.3	61.5	40.6‐79.8	1.86	88.9	33.3
>37.9	71.4	29.0‐96.3	57.7	36.9‐76.7	1.69	88.2	31.3
>41.9	85.7	42.1‐99.6	57.7	36.9‐76.7	2.03	93.8	35.3
**>45.0**	**100**	**59.0‐100.0**	**57.7**	**36.9‐76.7**	**2.36**	**100**	**38.9**

Abbreviations: CI, confidence interval; NPV, negative predictive value; PPV, positive predictive value.

Optimal cut‐off is donated in bold.

Using an increase in weight z‐score of > 0.21 points during this time produced a sensitivity and specificity of 100% and 59% respectively, and with a positive predictive validity of 100% and negative predictive validity of 40%.

### 3.4 Cytokine profile changes during EEN

Prior to EEN initiation, no differences in cytokine concentration were observed between responders and non‐responders. Using paired analysis, patients who entered clinical remission had a significant decrease in the concentration of IL‐6, IL‐17E, IL‐17F and IL‐31 (*P* < .05), (Table S3). Combining measurements from the start of EEN and end of EEN there were significant positive correlations found between FC and IFN‐y, IL‐1β, IL‐6, IL‐8, IL‐17a, IL‐17F, IL‐22, IL‐23, IL‐27, IL‐31 and IL‐33 in patients who achieved clinical remission, (Table S3). There was no change to cytokine levels in patients who did not achieve clinical remission.

### 3.5 FC changes during the early food reintroduction phase

A faecal sample was obtained at a median of 17 days (14, 21) post‐EEN in 23 patients of those who achieved remission to study changes in FC in the early food reintroduction phase. Within this early food reintroduction period, faecal calprotectin had significantly increased by 91% (2, 486) from their EEN cessation levels (54 days EEN: 430 mg/kg [141, 1047] vs 17 days post EEN 953 mg/kg [519, 1611]; *P* = .025) corresponding to a median daily rate of faecal calprotectin increase of 20 mg/kg/d (3.5, 50).

**Figure 2:**
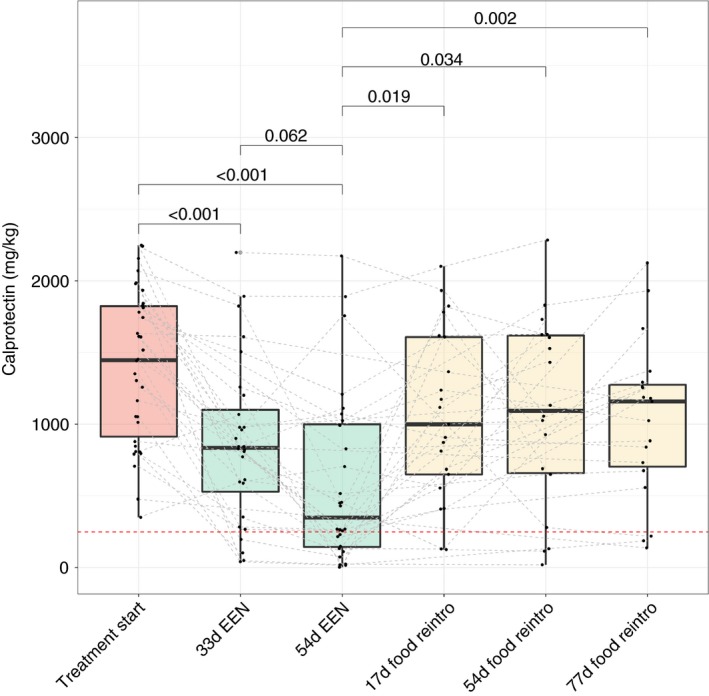
Faecal calprotectin concentrations during a course of EEN and at food reintroduction in patients entering clinical remission on EEN. Comparisons made between EEN start vs EEN End, and from EEN End till food reintroduction period; connecting lines indicating paired sample; reference line added at 250 mg/kg, indicating raised FC. *P* for Fisher pairwise comparisons following general linear model. Abbreviations: EEN, exclusive enteral nutrition

A second faecal calprotectin sample was collected at their first scheduled clinic review appointment. Due to the substantial variation in the timing these appointments took place at, participants and their associated collected samples (min: 30 days, max: 115 days) were divided into two groups; a medium‐term food (52 days [51, 57]); and a long‐term (72 days [65, 83]) food reintroduction phase. In the 20 patients whose samples were collected during the medium phase of food re‐introduction, faecal calprotectin concentration increased by 327 mg/kg (−295, 1345) compared with their end of EEN levels (52 days FC post EEN: 1094 mg/kg [660, 1625]; vs end of EEN: 430 mg/kg [141, 1047], *P* = .003). There was no difference in day 52 levels compared to baseline (pre‐EEN) (52 days FC: 1094 mg/kg [660, 1625] vs baseline FC: 1433 mg/kg [947, 1820], *P* = .124) (Figure 2). A faecal calprotectin sample was obtained from 19 other patients whose first post‐EEN clinic appointment was at a median of 72 days (65, 83) of food reintroduction. In these patients, faecal calprotectin had increased significantly from the end of EEN values (EEN end FC: 430 mg/kg [141, 1047] vs 72 days FC: 1159 mg/kg [676, 1293]; *P* < .001). However, there was no difference when compared to the early food reintroduction (*P* = 0.25) or medium food reintroduction (*P* = 0.19) (Figure 2). The increase in faecal calprotectin during food re‐introduction preceded changes to other disease activity markers (including wPCDAI, ESR and albumin) which remained similar to values at the end of EEN (Table S1).

### 3.6 MEN and changes in FC during food reintroduction

**Figure 3:**
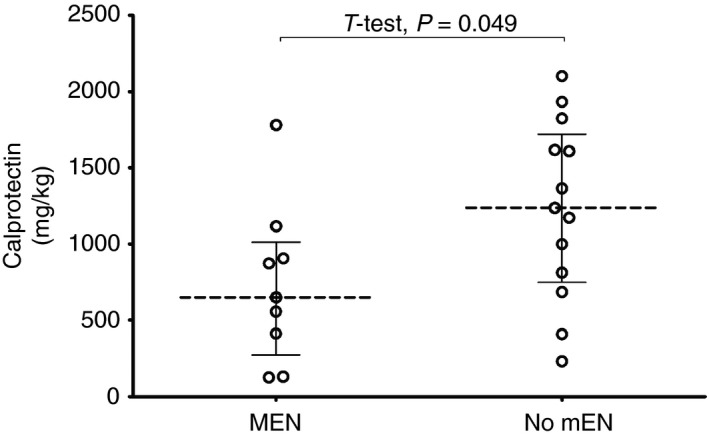
Faecal calprotectin concentrations during the early (17 d) food reintroduction period, grouping based on self‐reported MEN use. Dots representing individual values, line showing median and IQR. Abbreviations: MEN, Maintenance enteral nutrition

From the 41 patients who entered clinical remission, 29/42 (69%) were prescribed MEN upon EEN completion. The median prescribed volume of MEN was 400 mL (Q1: 262, Q3: 400), with a median prescribed energy intake of 450 Kcal/d (Q1: 323, Q3: 600) or 20% (15, 25) of patients daily EAR. Of the 37 food diaries returned, 15 (41%) recorded use of MEN. Patients who recorded use of MEN consumed a median of 333 Kcal [Q1: 330, Q3: 438] receiving 18% [Q1: 16.2, Q3: 24.7] of their recorded total energy intake (Table S4). Regardless of amount of MEN consumed, patients who recorded use of MEN during the early period of food reintroduction had significantly lower FC compared to those not using MEN (MEN users FC: 651 [271, 1781] vs non‐MEN users FC: 1238 [749, 2102], *P* = .049) (Figure 3).

In the same, early phase of food reintroduction, there was an inverse correlation between faecal calprotectin and MEN when expressed as volume (mL), calorific content (Kcal), % MEN contributed to TEI and % MEN contributed to patients EAR (MEN volume rho: −0.573, *P* = .041; MEN Kcal rho: −0.584, *P* = .036; MEN % TEI rho: −0.649, *P* = .016; MEN % EAR rho: −0.614, *P* = .026) (Figure S2). This effect was lost during the medium‐ and long‐term periods of food reintroduction.

### 3.7 Relapse rates at 1‐year post EEN

Twelve of the 41 (29%) patients who entered clinical remission on EEN, remained in remission 12 months post EEN. The median length of remission in patients who subsequently relapsed following completion of EEN was 207 days (Q1: 108, Q3: 328).

Survival analysis of MEN use, assessed either as prescribed by the clinical dietetic team, or according to their reported use on dietary records, was not associated with prolongation of length of remission (prescribed MEN group: 184 days [Q1: 103, Q3: 322] vs non prescribed MEN group: 257 days [167, 350]; *P* = .44; recorded MEN use: 181 days [71, 341] vs recorded non‐MEN use: 208 days [141, 212]; *P* = .70) (Figure S3). There was no difference in relapse rates at either 6 months (recorded MEN: 5/9 [56%] vs recorded non‐MEN: 10/32 [31%]; *P* = .18) or 12 months (recorded MEN: 8/9 [89%] vs recorded non‐MEN: 21/32 [66%]; *P* = .18) post‐EEN.

A faecal calprotectin cut‐off <100, <250 and <500 mg/kg was used in an attempt to explore the predictive ability of faecal calprotectin at the end of EEN with an increase in faecal calprotectin during food reintroduction. There was no difference in the subsequent rise in faecal calprotectin concentration during the early food reintroduction when using <100 mg/kg or <250 mg/kg as cut‐offs (FC<100 absolute change: 1158 [Q1: 612, Q3: 1615] vs FC >100 absolute change: 245 [−76, 699], *P* = .14; FC <250 absolute change: 612 [196, 1158] vs FC >250 absolute change: 245 [−157, 699], *P* = .23). Patients with a faecal calprotectin <500 mg/kg had a greater increase in faecal calprotectin both in absolute concentration and proportionally during early food reintroduction (FC <500 absolute change: 581 [182, 1206] vs FC >500 absolute change: 31 [−184, 284], *P *= .004; FC <500% change: 316 [81, 665] vs FC >500% change: 0.01 [−17, 27], *P* = .007).

During the medium food reintroduction, patients with faecal calprotectin <250 mg/kg and <500 mg/kg at the end of EEN had greater increases in faecal calprotectin both in absolute concentration (FC <250 absolute change: 1146 [82, 1620], vs FC >250 absolute change: 133 [−1117, 806], *P* = .04; FC <500: 1107 [82, 1466] vs FC >500: −281 [−1185, 394], *P* = .013) and proportionally (FC <250% change: 1024% [408, 21 521], vs FC >250% change: 20.8% [−61.4, 78.8], *P* = .008; FC <500% change: 541% [40, 3350] vs FC >500% change: −23.3% [−61.4, 25.8], *P* = .017). There was a similar increase, both in absolute concentration and proportional change in faecal calprotectin, when <100 mg/kg was used to stratify patients (FC <100:763 [24, 1571] vs FC >100:260 [−727, 1107], *P* = .29; FC<100:4787 [131, 46518] vs FC>100:26 [−56, 516], *P* = .08).

**Figure 4:**
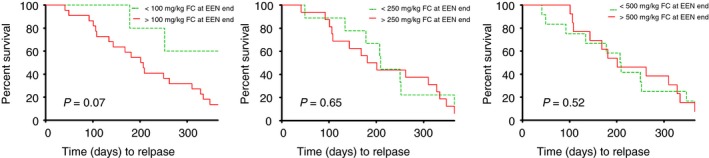
Kaplan‐Meier survival analysis using various cut‐offs in FC at the end of EEN to stratify patients with their time to subsequent relapse, censored at 1 y. FC, faecal calprotectin; EEN, Exclusive enteral nutrition.

Performing survival analysis using the concentration of faecal calprotectin at the end of EEN, neither a FC <250 mg/kg nor a FC <500 mg/kg value was associated with a longer time to subsequent clinical relapse (FC <250 remission length: 208 [145, 252] days, FC >250 remission length: 201 [107, 341] days; *P* = .41; FC <500 remission length: 230 [124, 320] days vs FC >500 remission length: 181 [108, 334] days; *P* = .59). When using the stricter cut‐off of FC <100 mg/kg at the end of EEN, there was a trend for patients to have longer periods of remission (FC <100 remission length: 382 days [215, 500] vs FC >100 remission length: 207 days [108, 334]; *P* = 0.07) (Figure 4).

### 3.8 Effect of other maintenance treatments

At the end of EEN (24/41, 59%), patients had begun the use of azathioprine as maintenance therapy. Rates of azathioprine use was similar between MEN and non‐MEN users (*P* = .17). Use of azathioprine at last follow‐up during food reintroduction was not associated with a significant increased remission length (Time to relapse, MEN users: 224 days [143, 334] days vs non‐MEN users: 180 days [78.5, 311], *P* = .51).

## 4 DISCUSSION

EEN continues to be the only established dietary treatment to induce remission in active paediatric Crohn's disease.^3^ It has the added benefit over corticosteroids of improving nutritional status and has higher rates of mucosal healing. The results of this study reflect those published in the literature, with the largest decrease in FC occurring within the first 4 weeks of treatment, and a slower yet persistent decrease during the second half of treatment.^8^, ^23^

The observed decrease in inflammatory cytokine expression is consistent with the published literature and reflects a reduction in systemic inflammation in patients responding to EEN in parallel with clinical activity improvement and reduction in faecal calprotectin.^25^

While, there was no difference in the levels of faecal calprotectin between patients achieving remission and those remaining with active disease at the end of EEN, this is in part due to the large interindividual faecal calprotectin response during EEN; as well as the faecal calprotectin remaining raised (>250 mg/kg) in 19/29 (66%) patients entering clinical remission at 8 weeks of EEN. Similar to treatment with corticosteroids, the levels of faecal calprotectin remain high in most patients who complete EEN, with this prospective study showing raised faecal calprotectin values indicative of ongoing colonic inflammation.^8^, ^23^, ^35^, ^36^, ^37^

It has also been shown that faecal calprotectin reverts to pre‐treatment levels within 4 months post‐EEN, and that up to 55% of patients in clinical remission can have a raised faecal calprotectin.^23^, ^38^ Complementing this previous work, this more detailed study demonstrated that the rise in FC post EEN to pre‐treatment levels occurs more rapidly than previously recognised.^23^ We show that a significant rise in faecal calprotectin is apparent within just 17 days of food reintroduction in the majority of patients and had reverted to pre‐treatment levels within 52 days of EEN cessation, despite concomitant maintenance treatment with azathioprine and/or MEN in most patients. This highlights the potent role of early dietary inflammatory triggers within the early food reintroduction phase in the post‐EEN period; as well as suggesting the maintenance therapies used are inadequate in preventing the development of subclinical inflammation following EEN.

The evidence to support the benefit of MEN to prolong remission following a successful course of EEN is at present inconsistent. The current study adds to the existing evidence by showing that MEN may delay the immediate rise of faecal calprotectin, during early food reintroduction; but this effect is only short‐lived and is probably at least partly dependent on the volume and calorific content of MEN consumed. This short‐term effect is likely to be due to displacement of part of the habitual diet by consuming MEN.^39^

The lack of short and long‐term compliance on MEN as reported in this study also highlights adherence challenges that patients with CD face and perhaps taste fatigue with MEN. There are many conflicting reports on the utility of MEN as a maintenance therapy. We have previously published a retrospective analysis showing up to 50% of patients who used MEN as their sole maintenance therapy remained in remission within a 1‐year follow‐up.^11^ This was similar to the response obtained in patients using azathioprine (azathioprine alone: 65%; azathioprine and MEN combined: 67%), and significantly better than those using no maintenance therapy (15%).

While the results from this earlier study suggested that MEN may be as effective as azathioprine in maintaining remission, it did not have a robust marker of compliance to MEN, as the current study did, making it difficult to assess patient's continued compliance during the length of the study, nor did it assess gut inflammation, as this study did. In this study using more detailed analysis, using patients’ recorded MEN use as well as measuring faecal calprotectin, we failed to replicate these previous results.

Another retrospective study, in which MEN was provided at 30% of EAR, also did not find the use of MEN to be associated with longer periods of remission, with 78% of patients who used MEN and 77% of patients who remained on habitual diet relapsing within 1 year.^18^ Of note however, EEN followed by MEN did have improved remission rates in the short term (6 months) and some of the patients in the study received MEN after a course of steroids rather than EEN.

The most compelling evidence to date for the use of MEN remains two RCTs in adults in Japan.^40^, ^41^ The first study compared MEN use against patients following their free habitual diet. The authors found MEN to be associated with a lower incidence of clinical relapse during one year of follow‐up.^40^ However, this study differs from this and previous reports as authors delivered MEN using elemental formula via nasogastric tube feeding, at 50% of patient's daily energy intake, at least twice as much as the current study. More recently, a second group from Japan have shown MEN at 50% using elemental formula to be as effective as 6‐mercaptopurine, in maintaining remission in a cohort of adult IBD patients in an RCT with a 2‐year follow‐up.^41^

Other groups have also reported on the clinical efficacy of MEN when recommended at high volumes of ~50% EAR.^42^, ^43^, ^44^ These observations in conjunction with the association we observed between the amount of consumed MEN and faecal calprotectin levels further support our hypothesis for a “dose‐response” association between MEN and disease relapse with the current study “under‐dosing” patients hence not demonstrating medium to long‐term clinical effect.

However, not all studies giving high dosage of MEN found prolonged length of remission. Knight et al (2005) gave patients with 1 L/d of MEN but without demonstrating a clinical benefit. However, the authors of this study did not formally record patient's compliance to MEN and felt this may have been a significant confounding factor with interpreting the results.^19^

The heterogeneity between studies and lack of a prospective RCT study in children remains a significant limitation in this area. While our study is not an RCT, we have attempted to prospectively study the effect of MEN while at the same time assessing its compliance with self‐reported weighted food diaries, a caveat not often captured within the existing literature.^11^, ^18^, ^45^, ^46^ With only half of the patients returning a food diary in which to assess MEN compliance this remains a limitation in our study but is useful to highlight many patients who are “prescribed” MEN do not actually take it, suggesting that better reinforcement from the treating team may help improve compliance.

As the beneficial effects of MEN in our study are associated with a reduced faecal calprotectin during the early period of food reintroduction only, when compliance is likely to remain highest, it remains unclear at present whether MEN can be used as a truly effective maintenance therapy in the longer term. This raises the question of developing other adjuvant dietary treatments with better tolerance and adherence profiles for long‐term dietary management of Crohn's disease.^47^ This has been supported by a recent study demonstrating patients prefer dietary advice rather than MEN after treatment with EEN.^48^ Such treatments can be used in isolation, interchangeably, or preferably in conjunction with MEN.

The mechanism of action for EEN, as with MEN, remains to be fully clarified, though several studies have implicated the importance of the gut microbiome.^49^, ^50^, ^51^, ^52^ To our knowledge there is only one study exploring the influence of MEN on the microbiome. However, as this was carried out in only two patients, with varying sample time‐points, it is difficult to place these results in the larger context, due to the inherent heterogeneity between subject's microbiome.^49^

Our data on clinical response to treatment are similar to previous reports but while patient's weight increased too, the magnitude of this effect was not as large as published historically.^5^, ^53^ This may be explained in part by our patients not having as low z‐scores suggesting improved detection and earlier diagnosis. The weight gain we observed in this study is similar  to other published literature.^5^, ^44^ We have demonstrated that changes in patient's weight during the first 4 weeks of treatment with EEN predicted which patients would enter clinical remission by the end of EEN. While this is very similar to the prediction validity using changes in faecal calprotectin during the same period, using weight change as a predictor creates a circular argument with assessing disease improvement using wPCDAI. We were unable to perform this analysis using changes in systemic markers of inflammation (CRP, ESR, inflammatory cytokines) as it is not the practice in our centre to routinely venepuncture patients at the midpoint of EEN, who are responding well clinically.

In conclusion, the current study confirms previous observations that EEN is a successful treatment in Crohn's disease inducing clinical remission and reducing FC. Exacerbation of subclinical inflammation is rapid and occurs soon after food reintroduction with MEN demonstrating a modest modifying effect only in the first few weeks. Future research should explore dietary triggers of Crohn's disease relapse during the food‐reintroduction phase and their interplay with the gut microbiome. The dose‐response signal identified in this study should be ascertained formally within well‐designed dose‐response studies.

## AUTHORSHIP

*Guarantor of the article*: Richard K Russell.

*Author contributions*: ML carried out and co‐ordinated most of the research activities and laboratory analysis, statistical analysis and produced the first draft for publication. CMC carried out patient recruitment, sample collection and part of the laboratory analysis. UZI contributed to the study design, ethical application, and funding award, and statistical analysis, and co‐supervised the researcher. LG phenotyped the patients identified and helped with patient follow‐up. HD helped with the dietary aspects and analysis. VG, LC, EB, TC, LA, CD, DMF, AB and RT identified and followed up patients. EM carried out part of the laboratory analysis. SM supervised the laboratory analysis and interpretation of results relating to the cytokine profile analysis, edited critically the first draft for publication. RH contributed to the study design, ethical application and funding award, edited critically the first draft for publication. KG conceived and developed the study design, ethical application, funding award, edited critically the first draft for publication, trained the researcher for laboratory analysis, co‐supervised the main researcher and co‐ordinated the study. RKR developed the study design, funding award, ethical application, identified and followed up patients, edited critically the first draft for publication and co‐supervised the researcher, trained the researcher in patient recruitment, co‐supervised the main researcher and co‐ordinated the study. All authors reviewed the final version of the manuscript and agreed to its content prior submission.
