# Supplementary figures and images for: The reduction of faecal calprotectin during exclusive enteral nutrition is lost rapidly after food re‐introduction
Source: Aliment Pharmacol Ther. 2019 Jul 25;50(6):664–74. doi: 10.1111/apt.15425 (PMC6772069; doi:10.1111/apt.15425)

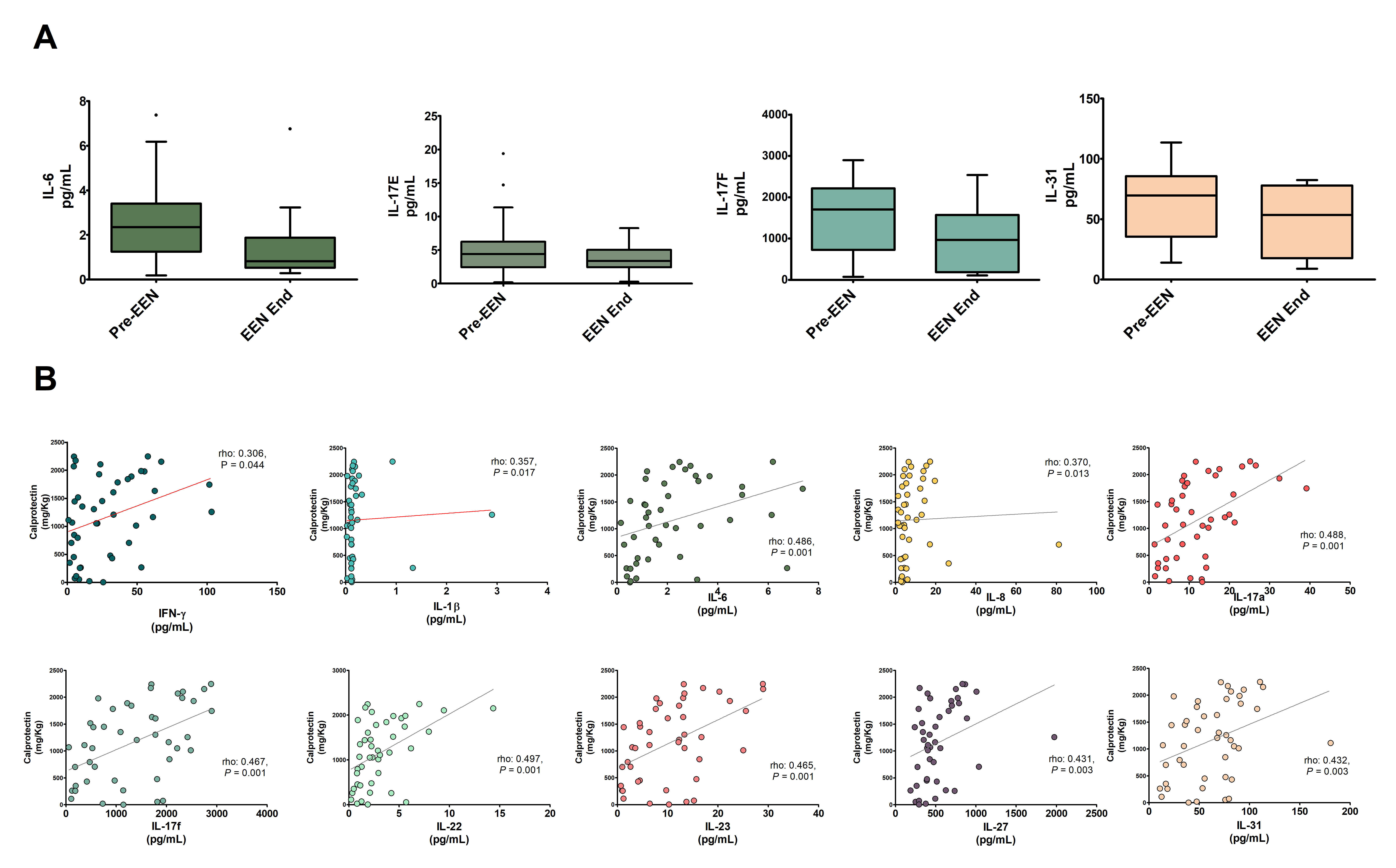

Supplement: Supplementary file 1 [file APT-50-664-s001.jpg]

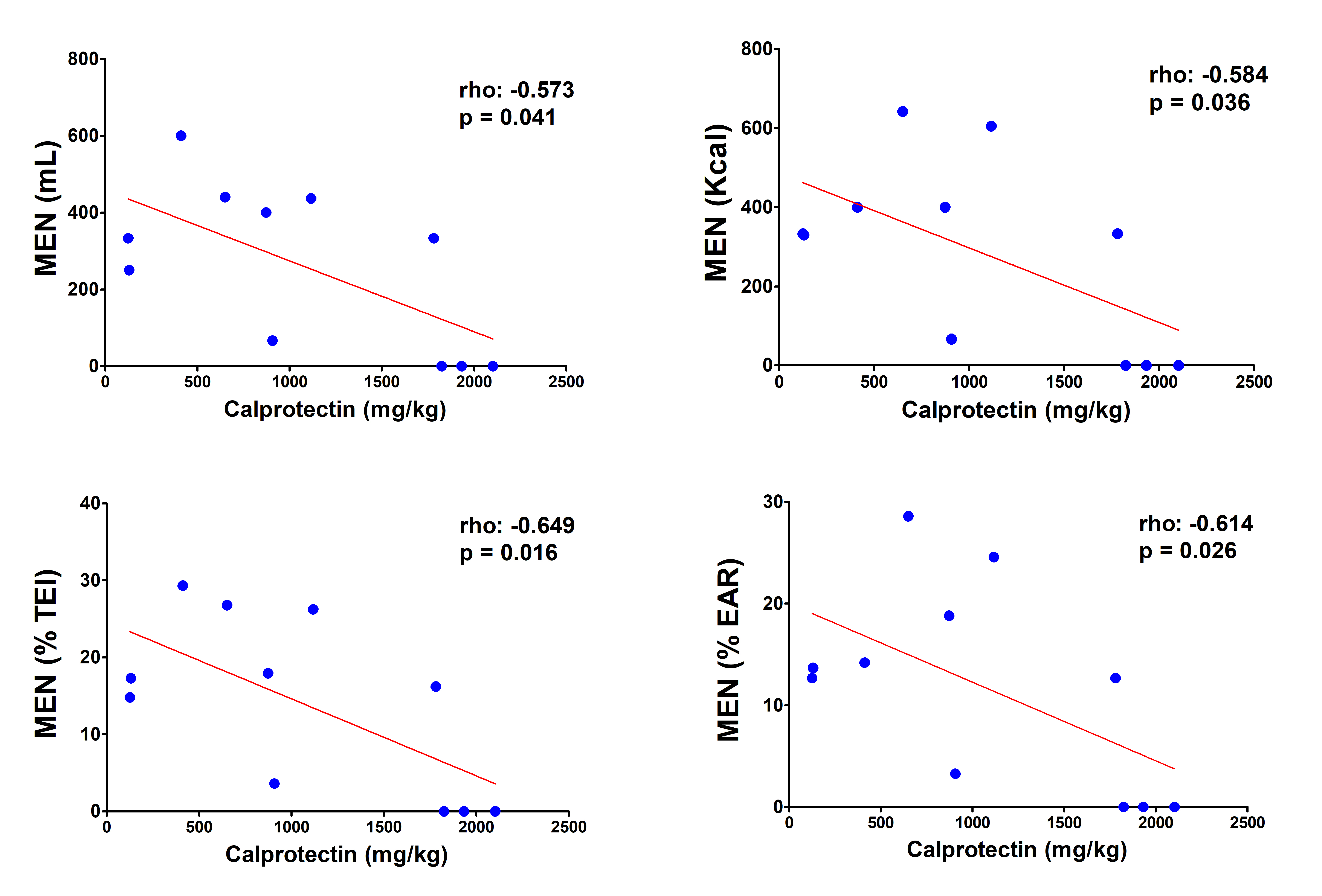

Supplement: Supplementary file 2 [file APT-50-664-s002.jpg]

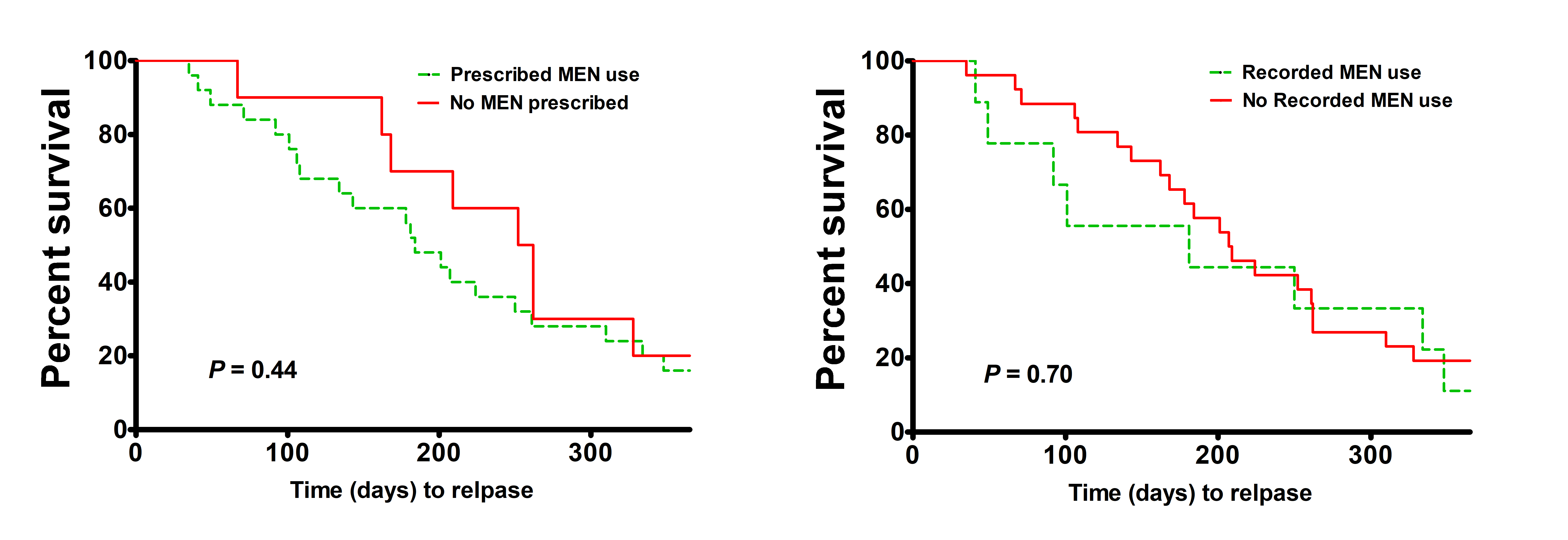

Supplement: Supplementary file 3 [file APT-50-664-s003.jpg]
