# Supplementary material for: The reduction of faecal calprotectin during exclusive enteral nutrition is lost rapidly after food re‐introduction
Source: Aliment Pharmacol Ther. 2019 Jul 25;50(6):664–74. doi: 10.1111/apt.15425 (PMC6772069; doi:10.1111/apt.15425)
Supplement: Supplementary file 4 [file APT-50-664-s004.docx]

Supplementary Table 1 Anthropometry & systemic inflammatory marker changes during EEN, and during food reintroduction

|  | Started EEN, but withdrew <8wk (n = 13) | | Completed 8wk EEN (n = 53) | | Clinically responded during 8wk EEN (n = 44) | | Achieve clinical remission on EEN (n = 41) | | | | |
| --- | --- | --- | --- | --- | --- | --- | --- | --- | --- | --- | --- |
|  | EEN Start | EEN End | EEN Start | EEN End | EEN Start | EEN End | EEN Start | EEN End | 17d food reintroduction | 52d food reintroduction | 72d food reintroduction |
| Weight (Kg) | 42.9 (35.1, 50.1) | 36.95 (32.3, 50.0) | 36.5 (27.8, 51.7) | 41.7 (29.1, 52.7) | 36.0 (28.1, 48.0) | 41.65 (29.0, 51.1) | 36.3 (28.9, 48.8) | 41.7 (30.0, 51.1) | 42.3(33.5, 52.5) | 43.5(34.9, 54.3) ^b^* | 40.0(32.8, 50.5) |
| Weight z-score | -1.3 (-2.6, -0.04) | -1.86 (-2.6, 0.08) | -0.40 (-1.32, 0.40) | -0.07 (-0.67, 0.32) ^a¥^ | -0.72 (-1.45, 0.01) | -0.01 (-0.68, 0.31) ^a¥^ | -0.64 (-1.40, 0.05) | -0.45 (-0.94, 0.17) ^a¥^ | -0.04 (-0.43, 0.65) | -0.17(-0.82, 0.29) | -0.17(-0.91, 0.05) |
| Height (m) | 1.58 (1.45, 1.65) | 1.51 (1.45, 1.60) | 1.52 (1.36, 1.65) | 1.51 (1.36, 1.66) | 1.51 (1.35, 1.62) | 1.51 (1.36, 1.63) | 1.52 (1.37, 1.62) | 1.51 (1.36, 1.63) | 1.50 (1.40, 1.60) | 1.60 (1.40, 1.70) | 1.51 (1.38, 1.64) ^b¥^ |
| Height z-score | -0.36 (-0.81, 0.37) | -1.15 (-1.4, 0.6) | -0.19 (-0.73, 0.56) | -0.34 (-0.87, 0.46) ^a^ | -0.29 (-0.85, 0.25) | -0.37 (-0.93, 0.21) ^a^ | -0.335 (-0.88, 0.24) | -0.45 (-0.94, 0.17) ^a^ | -0.34 (-0.75, 0.37) | -0.36 (-0.89, 0.20) | -0.33 (-0.98, 0.04) |
| BMI (kg/m^2^) | 16.5 (13.7, 19.9) | 16.7 (15.1, 19.0) | 16.7 (14.9, 18.5) | 18.0 (16.5, 19.8) | 16.3 (14.9, 17.9) | 18.0 (16.6, 19.8) | 16.65 (15.1, 18.0) | 18.0 (16.63, 19.6) | 18.3 (17.0, 20.1) | 17.9 (16.6, 19.9) | 17.2 (16.5, 19.4) |
| BMI z-score | -1.7 (-2.81, -0.48) | -1.69 (-2.25, 0.02) | -0.67 (-1.5, 0.3) | -0.04 (-0.46, 0.57) ^a¥^ | -0.7 (-1.66, 0.21) | 0.25 (-0.45, 0.59) ^a¥^ | -0.69 (-1.63, 0.21) | 0.03 (-0.45, 0.55) ^a¥^ | -0.01(-0.31, 0.68) | 0.045 (-0.76, 0.43) ^b^* | -0.08 (-0.75, 0.26) |
| ESR (mm/hr) | 30.5 (18.0, 42.5) | 28.0 (13.5, 61.5) | 16.0 (8.3, 32) | 8.5 (5.0, 17.5) ^a¥^ | 20.0 (10.3, 32.8) | 10.0 (5.0, 18.5) ^a¥^ | 19.0 (10.5, 32.5) | 9.5 (5.0, 17.3) ^a¥^ | 8.0 (2.75, 18.3) | 26.0 (9.0, 39.0) ^b^* | 29.0 (20.0, 41.8) ^b*^ |
| ↑ ESR (>20 mm/hr) | 7/9 | 4/5 | 19/44 | 8/36 | 18/36 | 7/29 | 16/33 | 6/26 | 2/10 | 9/15 | 5/6 |
| CRP (mg/L) | 15.0 (3.0, 48.5) | 7.0 (3.0, 21.0) | 6.0 (3, 22.8) | 2.5 (1.0, 3.0) ^a**^ | 9.0 (3.0, 23.3) | 2 (1.0, 3.0) ^a**^ | 9.0 (3.0, 22.0) | 2.0 (1.0, 3.0) ^a**^ | 2.0 (1.0, 3.0) | 4.0 (3.0, 15.0) ^b*^ | 6.5 (3.0, 11.8) ^b*^ |
| ↑ CRP (>7mg/L) | 6/9 | 1/3 | 22/48 | 5/34 | 20/38 | 3/27 | 18/35 | 3/24 | 0/11 | 6/15 | 3/6 |
| Albumin (g/L) | 30.5 (25.8, 36.0) | 35.5 (30.5, 36.8) | 35.0 (27.0, 38.0) | 38.0 (37.0, 40.5) ^a¥^ | 34.0 (26.5, 37.5) | 38.0 (37.0, 40.3) ^a¥^ | 34.5 (27.0, 37.3) | 38.0 (37.0, 41.0) ^a¥^ | 37.0 (33.5, 40.0) ^b^* | 37.0 (33.0, 40.3) | 36.0 (31.5, 39.0) ^b*^ |
| ↓ Albumin (<35 g/L) | 6/9 | 1/4 | 23/51 | 2/37 | 21/41 | 2/30 | 19/38 | 2/27 | 4/13 | 6/18 | 4/9 |
| Haemoglobin (g/L) | 109.0 (95.0, 121.0) | 104.0 (90.5, 127.5) | 124.0 (112.0, 129.8) | 120.5 (110.0, 127.5) ^a^ | 124.0 (112.0, 130.5) | 121.0 (113.0, 130.0) ^a^ | 124.0 (112.0, 132.0) | 121.5 (113.3, 129.0) ^a^ | 120.0 (115.5, 128.5) | 128.0 (118.0, 135.0) | 127.0 (114.0, 134.5) |
| ↓ Haemoglobin | 6/8 | 4/6 | 22/48 | 18/38 | 19/38 | 14/31 | 18/35 | 12/28 | 4/12 | 5/19 | 2/9 |
| wPCDAI | 57.5 (45.0, 65.0) | 37.5 (35.0, 55.0) | 32.5 (21.25, 55.0) | 7.5 (0, 10.0) ^a¥^ | 40.0 (22.5, 57.5) | 0.0 (0.0, 7.5) ^a¥^ | 35.0 (20.6, 56.3) | 0.0 (0.0, 7.5) ^a¥^ | 0.0 (0.0, 21.3) | 7.5 (0.0, 16.9) ^b^* | 5.0 (0.0, 19.4) ^b*^ |
| Calprotectin wet matter (mg/kg) | 1247 (892, 1580) | 1438.7 (1227, 1445) | 1452 (946, 1835) | 453 (165, 1100) ^a¥^ | 1518 (946, 1888) | 440 (165, 1059) ^a¥^ | 1452 (880, 1843) | 430 (141, 1047) ^a¥^ | 953 (519, 1611) | 1094 (660, 1625) | 1159 (676, 1293) ^b**^ |
| Calprotectin dry matter (mg/kg) | 10335 (3558, 14830) | 7468 (6138, 9591) | 8221 (5032, 11266) | 2247 (652, 5414) ^a¥^ | 8509 (5032, 11561) | 1948 (652, 5096) ^a¥^ | 8509 (4980, 11414) | 1686 (596, 4997) ^a¥^ | 4085 (1791, 7612) | 3992 (1072, 6439) | 3491 (1931, 4969) |
| Data are displayed with medians and interquartile range. Calprotectin expressed per wet faecal matter, and as dry faecal matter (accounting for dilution effect due to increased moisture content at baseline). a significant difference between EEN start vs EEN End; b significant difference between EEN End; * P < 0.05; ** P < 0.01; ¥ P < 0.001. P for Fisher pairwise comparisons following general linear model. Abbreviations: BMI, body mass index; ESR, erythrocyte sedimentation rate; CRP, C-reactive protein; wPCDAI, weighted pediatric Crohn's disease activity index | | | | | | | | | | | |

Supplementary Table 2 Remission rates based on disease location.

|  | Colonic | Ileal | Ileocolonic | Upper Oesophageal | All |
| --- | --- | --- | --- | --- | --- |
| No Remission | 11 (9.5) | 4 (2.3) | 10 (12.9) | 0 (0.38) | 25 |
| Achieved remission | 14 (15.5) | 2 (3.7) | 24 (21.1) | 1 (0.6) | 41 |
| All | 25 | 6 | 34 | 1 | 66 |
| Counts of those patient’s entering remission (Expected count) | | | | | |

Supplementary Table 3 Plasma cytokine concentrations during EEN

|  | EEN Start | EEN End | Correlation between FC and cytokines |
| --- | --- | --- | --- |
| IFN-y | 29.7 (8.4, 53.5) | 9.7 (5.5, 32.2) | 0.31* |
| IL10 | 0.27 (0.18, 0.37) | 0.46 (0.24, 0.91) | 0.05 |
| IL12p70 | 0.2 (0.1, 0.3) | 0.14 (0.10, 0.20) | 0.16 |
| IL13 | 3.3 (2.6, 6.2) | 3.6 (2.0, 4.1) | 0.02 |
| IL1β | 0.1 (0.1, 0.2) | 0.1 (0.1, 0.1) | 0.49 * |
| IL2 | 0.3 (0.3, 0.6) | 0.4 (0.3, 0.7) | 0.13 |
| IL4 | 0.02 (0.02, 0.03) | 0.02 (0.02, 0.10) † | -0.18 |
| IL6 | 2.4 (1.3, 3.4) | 0.8 (0.4, 1.2) * | 0.49 ^¥^ |
| IL8 | 5.3 (3.5, 11.0) | 3.9 (2.8, 13.8) | 0.37 ** |
| TNF-α | 1.7 (1.3, 2.2) | 1.7 (1.5, 2.1) | 0.15 |
| IL17a | 11.7 (6.6, 15.7) | 8.3 (4.2, 18.9) | 0.49 ^¥^ |
| IL17E | 4.4 (2.5, 6.6) | 3.4 (2.7, 5.1) ** | 0.13 |
| IL17F | 1745 (626, 2245) | 968 (186, 1409) * | 0.47 ^¥^ |
| IL21 | 1485 (870, 1793) | 772 (4, 1803) | 0.22 |
| IL22 | 2.8 (2.0, 5.6) | 1.3 (0.8, 2.9) † | 0.5 ^¥^ |
| IL23 | 13.0 (4.5, 15.9) | 6.5(1.2, 12.3) † | 0.47 ^¥^ |
| IL27a | 467.5 (379.0, 757.0) | 389.1 (294.1, 626.4) † | 0.47 ^¥^ |
| IL31 | 70.5 (34.5, 85.7) | 53.4 (18.5, 78.5) * | 0.43 ^¥^ |
| IL33 | 4.5 (1.6, 6.5) | 2.4 (0.4, 5.5) † | 0.47 ^¥^ |

Descriptive statistics (median [IQR]) of cytokine concentrations (pg/mL) at EEN start and EEN end. Paired t test indicating significant change in cytokine concentration during EEN among patients entering clinical remission. Spearman rank correlation between pooled FC and cytokine measurements from baseline and end of EEN. P value significance indicated: † P < 0.1; ^*^ P < 0.05; ^**^ P < 0.01; ^¥^ P < 0.001

Supplementary Table 4 Descriptive statistics of maintenance enteral nutrition use as per prescribed dietic record, and patient self-reported food diary. Paired statistics between prescribed vs reported use

|  | Median (IQR) |  |
| --- | --- | --- |
| MEN prescribed volume (mL) | 400 (310, 410) ** |  |
| MEN prescribed (kcal) | 420 (345, 600) ** |  |
| MEN reported volume (mL) (3-day record) | 333 (250, 400) |  |
| MEN reported (kcal) (3-day record) | 333 (330, 438) |  |
| % MEN Kcal / TEI (3-day record) | 18 (16, 25) |  |
| % MEN Kcal / EAR (3-day record) | 16 (13, 25) |  |
| Paired t test indicating difference between prescribed and patient reported use.  ^*^ P < 0.05; ^**^ P < 0.01; ^¥^ P < 0.001 | |  |
